# An anesthetic experience with remimazolam for MELAS patients

**DOI:** 10.1186/s40981-022-00550-3

**Published:** 2022-08-06

**Authors:** Atsuhiro Kitaura, Shinichi Nakao

**Affiliations:** 1 Department of Anesthesiology, Kindai University Faculty of Medicine, Osaka-Sayama, Japan; 2 Okanami General Hospital, Iga-Ueno, Mie Japan

**Keywords:** Remimazolam, Anesthesia, MELAS

Letter to Editor

In reply,

We read with interest Dr. Finsterer’s letter-to-editor [1] about our study [2]. We appreciate the expert comments. We agree that there were some shortcomings in our current study.

First, it is true that metabolic acidosis develops because of elevation of serum lactate. We made a mistake. Second, in our case, we did not present the heteroplasmy rate because it was never tested in his medical history. We agree that the heteroplasmy rate in various tissues is important to know their clinical course and prognosis [3]. We do not deny this point, but as anesthesiologists, it is somewhat difficult to deal with heteroplasmy rates. Because it is a progressive, untreatable genetic disease, a certain number of patients are actually managed without testing for heteroplasmy rates such as our patient. In addition, access to information regarding heteroplasmy rates is often difficult in the anesthesia setting at surgical facilities. Moreover, there are no reports that adequately demonstrate the extent to which heteroplasmy rates affect the pharmacological effects of anesthetics. Third, enough medical information was not included in our previous case report, due to the limitation of the word count. Our case had no history of anesthesia prior to MitraClip®. His medications included carvedilol, lansoprazole, lanthanum carbonate hydrate, candesartan, amedinium methyl sulfate, olanzapine, and lorazepam. Our patient's muscle strength and tendon reflexes were diminishing as the myopathy progresses. The residual pathological tendon hyperreflexia was probably due to his brain atrophy, which had been present since the diagnosis of MELAS.

Propofol is the most prevalent intravenous anesthetic and safely used in an ordinary clinical setting even for patients with mitochondrial dysfunction including MELAS syndrome [4, 5], even though propofol is demonstrated to weakly suppress the respiratory chain in the mitochondria in vitro studies [6]. Propofol infusion syndrome (PRIS) is a quite rare but lethal complication of propofol [7–9]. Neither exact pathophysiology nor clear risk factors of the PRIS has been elucidated, and an animal model of the PRIS has not been made. Right now, serious mitochondrial dysfunction induced by propofol overdoses is the most reliable pathophysiology of the PRIS. Therefore, we stated that propofol can be safely used in patients with MELAS if you use propofol only for induction of general anesthesia or even maintenance of general anesthesia using appropriate doses, but we believe that it is safer to avoid the continuous administration of propofol in patients with mitochondrial disorders, because the continuous administration sometimes leads to overdoses, and in addition we can perform general anesthesia without propofol using other general anesthetics.

Remimazolam is a benzodiazepine derivative, and another benzodiazepine derivative, midazolam, is widely recognized as a safe sedative in a patient with MELAS syndrome [5, 10]. That is why we used remimazolam and this is the first report that remimazolam was safely used in a patient with MELAS syndrome for general anesthesia. It is true that our paper is only a case report, and we understand that further appropriately designed studies should be required to warrant the safety of remimazolam for MELAS patients.

## References

[1] Finsterer J (2022). Assessing the anesthetic effectiveness of remimazolam in MELAS patients requires careful investigations. JA Clin Rep.

[2] Kitaura A, Kosumi R, Iwamoto T (2022). Remimazolam anesthesia for transcatheter mitral valve repair in a patient with mitochondrial myopathy, encephalopathy, lactic acidosis, and stroke-like episodes (MELAS) syndrome: a case report. JA Clin Rep.

[3] Shi Y, Chen G, Sun D, Hu C, Liu Z, Shen D, Wang J, Song T, Zhang W, Li J, Ren X, Han T, Ding C, Wang Y, Fang F (2022). Phenotypes and genotypes of mitochondrial diseases with mtDNA variations in Chinese children: a multi-center study. Mitochondrion..

[4] Footitt EJ, Sinha MD, Raiman JA, Dhawan A, Moganasundram S, Champion MP (2008). Mitochondrial disorders and general anaesthesia: a case series and review. Br J Anaesth..

[5] Gurrieri C, Kivela JE, Bojanic K, Gavrilova RH, Flick RP, Sprung J (2011). Anesthetic considerations in mitochondrial encephalomyopathy, lactic acidosis, and stroke-like episodes syndrome: a case series. Can J Anaesth..

[6] Sproule DM, Kaufmann P (2008). Mitochondrial encephalopathy, lactic acidosis, and strokelike episodes: basic concepts, clinical phenotype, and therapeutic management of MELAS syndrome. Ann NY Acad Sci..

[7] Bray RJ (1998). Propofol infusion syndrome in children. Paediatr Anaesth.

[8] Wolf A, Weir P, Segar P, Stone J, Shield J (2001). Impaired fatty acid oxidation in propofol infusion syndrome. Lancet..

[9] Mirrakhimov AE, Voore P, Halytskyy O, Khan M, Ali AM (2015). Propofol infusion syndrome in adults: a clinical update. Crit Care Res Pract..

[10] Rafique MB, Cameron SD, Khan Q, Biliciler S, Zubair S (2013). Anesthesia for children with mitochondrial disorders: a national survey and review. J Anesth..

